# Risk Factors for Developing Hyponatremia in Thyroid Cancer Patients Undergoing Radioactive Iodine Therapy

**DOI:** 10.1371/journal.pone.0106840

**Published:** 2014-08-29

**Authors:** Jung Eun Lee, Seung Kyu Kim, Kyung Hwa Han, Mi Ok Cho, Gi Young Yun, Ki Hyun Kim, Hoon Young Choi, Young Hoon Ryu, Sung Kyu Ha, Hyeong Cheon Park

**Affiliations:** 1 Department of Internal Medicine, Yongin Severance Hospital, Yonsei University College of Medicine, Yongin, Republic of Korea; 2 Department of Internal Medicine, Gangnam Severance Hospital, Yonsei University College of Medicine, Seoul, Republic of Korea; 3 Department of Nuclear Medicine, Gangnam Severance Hospital, Yonsei University College of Medicine, Seoul, Republic of Korea; 4 Biostatistics Collaboration Unit, Gangnam Medical Research Center, Seoul, Republic of Korea; 5 Severance Institute for Vascular and Metabolic Research, Yonsei University College of Medicine, Seoul, Republic of Korea; University of Michigan, United States of America

## Abstract

**Background:**

Due to the alarming increase in the incidence of thyroid cancer worldwide, more patients are receiving postoperative radioactive iodine (RAI) therapy and these patients are given a low-iodine diet along with levothyroxine withdrawal to induce a hypothyroid state to maximize the uptake of RAI by thyroid tissues. Recently, the reported cases of patients suffering from life-threatening severe hyponatremia following postoperative RAI therapy have increased. This study aimed to systematically assess risk factors for developing hyponatremia following RAI therapy in post-thyroidectomy patients.

**Methods:**

We reviewed the medical records of all thyroid cancer patients who underwent thyroidectomy and postoperative RAI therapy from July 2009 to February 2012. Demographic and biochemical parameters including serum sodium and thyroid function tests were assessed along with medication history.

**Results:**

A total of 2229 patients (47.0±11.0 years, female 76.3%) were enrolled in the analysis. Three hundred seven patients (13.8%) of all patients developed hyponatremia; 44 patients (2.0%) developed moderate to severe hyponatremia (serum Na^+^≤130 mEq/L) and another 263 (11.8%) patients showed mild hyponatremia (130 mEq/L<serum Na^+^≤135 mEq/L). In univariate analysis, old age, female sex, presence of hypertension, presence of diabetes, use of thiazide diuretics, use of angiotensin receptor blocker or angiotensin-converting enzyme inhibitors, lung metastasis, and hyponatremia and lower estimated glomerular filtration rate at the start of RAI therapy were significantly associated with hyponatremia in patients undergoing RAI therapy after total thyroidectomy. Multivariate analysis showed that old age, female sex, use of thiazide diuretics, and hyponatremia at the initiation of RAI therapy were independent risk factors for the development of hyponatremia.

**Conclusion:**

Our data suggest that age greater than 60 years, female sex, use of thiazide, and hyponatremia at the initiation of RAI therapy are important risk factors for developing hyponatremia following RAI therapy in post-thyroidectomy patients.

## Introduction

In the last decades, the incidence of thyroid cancer has increased at an alarming rate worldwide [1]. Differentiated thyroid cancer (DTC) accounts for the vast majority of thyroid cancers and initial treatment includes ablative radioactive iodine (RAI) therapy after thyroidectomy [2]. Long-term comprehensive studies have demonstrated that ablative RAI therapy decreases the rates of regional recurrences and disease-associated mortality [3]. Many centers use treatment protocols that include thyroid hormone withdrawal and 2–4 weeks of a low-iodine diet prior to RAI therapy to minimize dietary iodine interference and induce hypothyroid status for facilitating the uptake of RAI [3]. Iatrogenic hypothyroid status induced by such treatment protocol may impair water excretion and cause mild hyponatremia. Such a low-iodine diet protocol is frequently accompanied by low dietary salt intake. In addition, these patients are encouraged to increase oral fluid intake during RAI therapy to flush out the iodine. Thus, the disturbances in the serum sodium concentration may be further aggravated by low dietary salt and increased oral fluid intake during RAI therapy.

Until now, few studies have investigated the incidence and severity of hyponatremia in hypothyroid patients following RAI therapy after thyroidectomy. A previous retrospective analysis of patients who underwent thyroid-ablation in the setting of thyroid hormone withdrawal performed by Baajafer el al. demonstrated that only few acute hypothyroid patients experienced hyponatremia and none of the patients had severe symptomatic hyponatremia [4]. The same authors also reported in a prospective study of 212 thyroid cancer patients undergoing RAI therapy that clinically-important hyponatremia was uncommon [5]. However, there have been a few case reports of severe symptomatic hyponatremia in acute hypothyroid patients undergoing RAI therapy [6]–[8]. We also recently experienced life-threatening severe hyponatremia in two patients at our center [9].

Thus, the aim of this study was to systematically evaluate the incidence and risk factors for development of hyponatremia after RAI therapy in thyroid cancer patients who underwent total thyroidectomy.

## Materials and Methods

### Study population

Data for a total of 2241 DTC patients who consecutively underwent bilateral total thyroidectomy with central compartment neck dissection and RAI therapy at Gangnam Severance hospital were collected from July 2009 to February 2012. We excluded 12 patients due to absence of the complete set of laboratory findings for serum sodium, free T4 (fT4), or thyroid stimulating hormone (TSH). Thus, the data for 2229 patients were analyzed. All of the patients were treated using the same RAI therapy protocol of our thyroid cancer center. Briefly, after thyroidectomy, the patients took levothyroxine daily for four weeks and then they were switched to liothyronine daily for another two weeks. After that, the patients were advised to follow a low iodine diet along with thyroid hormone withdrawal for two weeks prior to RAI therapy. This study was approved by the Ethics Committee of the Gangnam Severance Hospital (#3-2014-0039). A written informed consent was not necessary because this was a retrospective cohort study.

### Laboratory data

Baseline blood tests, including renal function screening and serum electrolyte testing, were performed on the day of admission for RAI therapy. Serum sodium concentrations were obtained at baseline and at the time when the patients were cleared for discharge or upon the development of clinical symptoms. The following demographic data were collected; age, sex, use of anti-hypertensive medications such as thiazide or angiotensin receptor blocker (ARB) or angiotensin-converting enzyme inhibitors (ACEi), presence of co-morbidities such as diabetes or hypertension, and presence of lung or brain metastasis. After we identified baseline characteristics of all the patients, we divided them into three groups according to the serum sodium level as follows: (1) the moderate to severe hyponatremia group, in which the lowest serum sodium level within seven days after RAI therapy was less than 130 mEq/L (n = 44, 2.0%), (2) the mild hyponatremia group, in which the serum sodium level was 131∼135 mEq/L (n = 263, 11.8%), and (3) the normonatremic group, in which the lowest serum sodium level within seven days after RAI therapy was more than 136 mEq/L (n = 1922, 86.2%). Serum sodium and creatinine concentrations were determined using an Olympus AU 2700 analyzer. The normal range of serum sodium is 136–146 mEq/L. Serum TSH and free thyroxine concentrations were determined using the Abbott TSH assay, free thyroxine assay kits, and an Abbott Architect i1000 analyzer. The normal ranges for fT4 and TSH are 0.7–1.48 ng/dL and 0.35–4.94 mcIU/mL, respectively. The estimated glomerular filtration rate (eGFR) was calculated using the simplified Modification of Diet in Renal Disease formula (MDRD) and chronic renal failure was defined as an eGFR less than 60 ml/min/1.73 m^2^. Also, the decrease rate in the eGFR during RAI therapy was calculated using the following formula: eGFR decrease rate = (pre-RAI therapy eGFR−post-RAI therapy eGFR)÷pre-RAI therapy eGFR.

### Statistical analysis

Continuous variables are presented as mean ± SD, and categorical variables as numbers and percentages. Baseline characteristics of the patients were compared using the student’s *t* test for continuous variables and χ^2^ test for categorical variables. We performed univariate and multivariate logistic regression analyses to determine the association between clinical variables and hyponatremia following RAI therapy in patients with thyroid cancer. Prior to conducting logistic regression analysis to determine the influence of each variable on susceptibility to developing hyponatremia after RAI therapy, we confirmed that the proportion of patients diagnosed with hypertension increased with age when estimated by linear association and all of the thiazide medication users had hypertension. Therefore, hypertension was excluded from the variables because multicollinearity could occur between hypertension, old age (greater than 60 years), and thiazide medication use in the logistic regression analysis. P-values less than 0.05 were considered to be statistically significant. All statistical analyses were performed using SAS version 9.2 (SAS Institute Inc. Cary, NC, USA).

## Results

### Study population

A total of 2,229 patients (76.3% females, mean age 47.0±11.0 years) were included in this study. Among them, 97% of patients had papillary thyroid cancer. In the total 2,229 patients, 15.8% of patients were more than 60 years of age, and 13.8% of patients had hyponatremia. Among them, 8 (0.35%) patients showed severe hyponatremia and the serum sodium level was less than 120 mEq/L following RAI therapy. Thirty patients (1.3%) had underlying chronic renal failure, which was defined as eGFR less than 60 ml/min/1.73 m^2^. Fifteen percent of the patients had hypertension, and six percent of patients had diabetes. Four percent of all patients used thiazide diuretics, and nine percent of all patients took ARB or ACEi. Twenty-three patients (1.0%) had lung metastasis, but none of the patients had brain metastasis.

**Figure 1:**
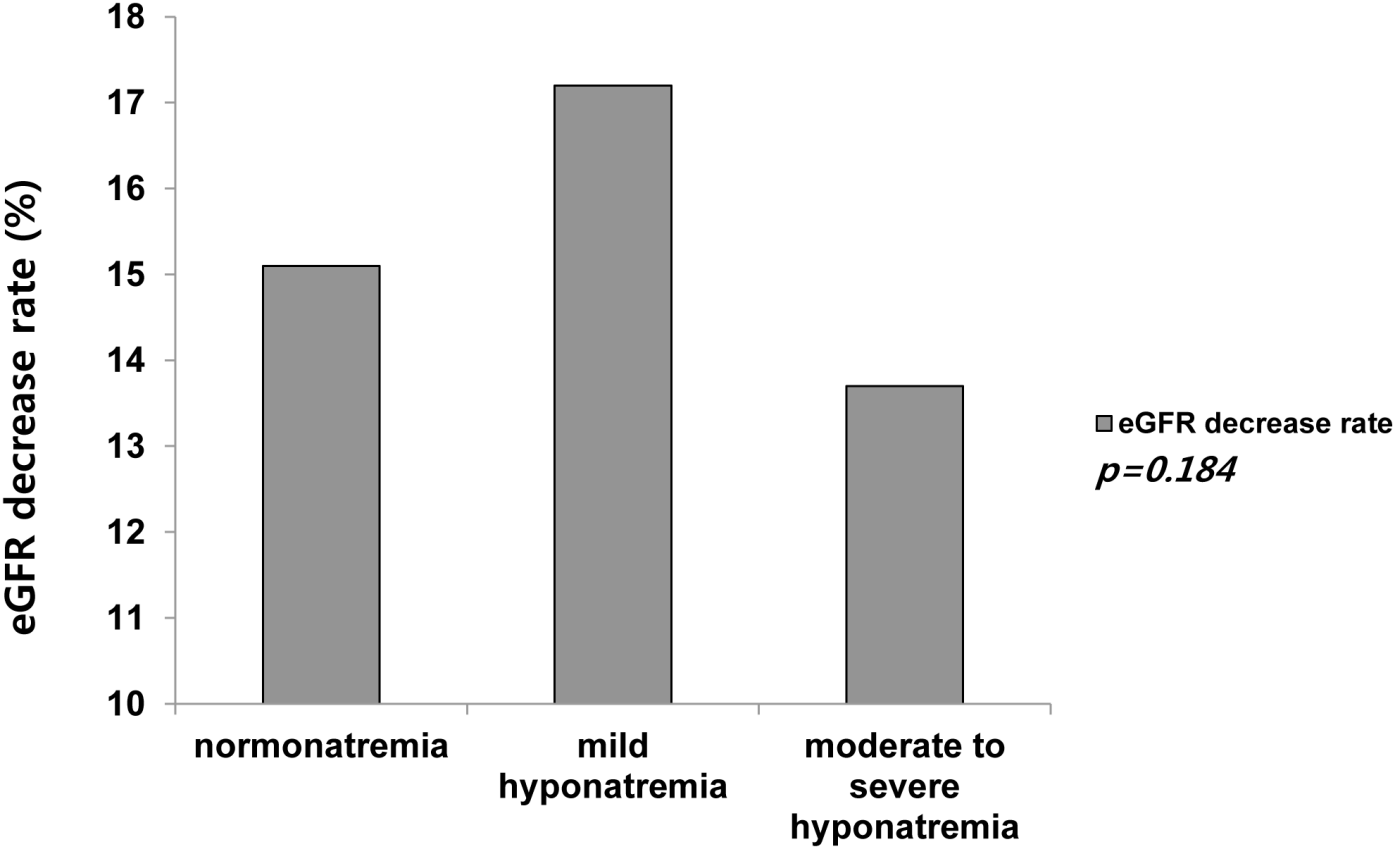
Comparison of eGFR decrease rate between pre- and post- RAI therapy.

When parameters were compared between the normonatremia, mild hyponatremia, and moderate to severe hyponatremia groups, statistically significant differences were found in age, sex, presence of hypertension or diabetes, use of thiazide or ARB medications, lung metastasis, serum sodium, calcium, and albumin. These results are summarized in Table 1. Compared with the patients in the normonatremia group, patients with varying degrees of hyponatremia were older and suffered from hypertension or diabetes more frequently, had lung metastasis more frequently, or had used thiazide or ARB more frequently. There were greater number of females among all of the hyponatremic patients belonging to the moderate to severe hyponatremia and mild hyponatremia groups than in the normonatremia group (81.7% vs. 75.4%, p = 0.016). The mean serum sodium and eGFR at the start of RAI therapy in the patients of the moderate to severe hyponatremia and mild hyponatremia groups were lower than those in the patients of the normonatremia group. Serum fT4, T3, and TSH levels were not significantly different among three groups. The decrease rate in the eGFR following RAI therapy was not significantly different among three groups according to serum sodium levels (p = 0.184) (Fig. 1).

**Table 1:** Baseline characteristics of patients and changes in various laboratory findings according to the sodium status following RAI therapy.

	Moderate to severe Hyponatremia(n = 44)	Mild hyponatremia(n = 263)	Normo-natremia(n = 1922)	P value
Age (years)	61.9±11.2	49.1±13.8	46.5±11.3	<0.0001
Age ≥60 years, n (%)	27(61.3)	65(24.7)	262(13.6)	<0.0001
Female sex, n (%)	32(72.7)	219(83.2)	1449(75.4)	0.016
Hypertension, n (%)	24(54.5)	59(22.4)	255(13.2)	<0.0001
DM, n (%)	5(11.3)	30(11.4)	103(5.3)	0.002
Thiazide use, n (%)	11(25.0)	24(9.1)	67(3.5)	<0.0001
ARB or ACEi use, n (%)	10(22.7)	41(15.5)	157(8.2)	0.002
Lung metastasis, n (%)	3(6.8)	4(1.5)	16(0.8)	0.005
Laboratory findings before RAItherapy				
fT4	0.14±0.09	0.12±0.05	0.12±0.07	0.245
T3 (ng/dL)	24.9±10.3	23.1±7.7	23.3±8.9	0.453
TSH (mcIU/mL)	79.7±21.8	81.3±20.9	79.2±20.4	0.300
Na (mEq/L)	137.1±3.6	138.8±2.0	139.9±1.8	0.000
Cr (mg/dL)	0.97±0.37	0.90±0.25	0.89±0.23	0.111
eGFR (ml/min/1.73 m^2^)	75.05±23.01	81.26±22.74	83.40±22.02	0.019
Ca (mg/dL)	9.17±0.72	9.21±0.63	9.10±0.63	0.027
Albumin (g/dL)	4.43±0.44	4.50±0.28	4.54±0.27	0.003

Note: values for categorical variables are expressed as number (percentage); values for continuous variables are expressed as mean ± standard deviation.

Abbreviations: DM, diabetes mellitus; ARB, angiotensin receptor blocker; ACEi, angiotensin converting enzyme inhibitor; RAI, radioactive iodine; TSH, thyroid stimulating hormone; eGFR, estimated glomerular filtration rate.

### In-depth findings in moderate to severe hyponatremic patients

The medical records of moderate to severe hyponatremic patients (n = 44, females 32) were reviewed in detail to investigate the clinical characteristics and possible risk factors for developing clinically significant hyponatremia after RAI therapy. Among these 44 patients, 11 patients had used thiazide diuretics, 10 patients had used ARB or ACEi, and 3 patients had lung metastasis. The serum creatinine level in one female patient was 2.9 mg/dL and the serum creatinine levels in the remaining patients were less than 1.5 mg/dL when they started RAI therapy. At that time, fourteen patients already had decreased renal function, which was defined as an eGFR less than 60 ml/min/m^2^. All of the 44 patients had nausea or vomiting following RAI therapy and needed to visit the outpatient clinic or the emergency room. Eight of these 44 patients showed severe hyponatremia, which was defined as serum sodium level less than 120 mEq/L (mean 116 mEq/L, range 108∼119 mEq/L). All of these eight patients were females and they were more than 60 years of age, excluding one female patient, who was 47 years old. Six of these patients had hypertension; 3 patients took ARB or ACEi, and 3 patients took thiazide medication. The type of thyroid cancer was papillary cancer in all patients, excluding one patient, who had follicular carcinoma. None of the patients had metastasis to the other sites. All of the patients who had nausea or vomiting visited the emergency room within an average 2.3 days (range, 1∼4 days) following RAI therapy. The plasma osmolarity of all patients with available data were decreased indicating presence of true hyponatremia. Most of urine osmolarity values were greater than 100 mosm/kg that indicates an impairment of free water excretion. The urine sodium level of these patients were rather low suggesting possible dehydration due to nausea and vomiting (Table 2).

**Table 2:** Eight patients with severe hyponatremia following radioactive iodine therapy.

Patient	Age(year)/Sex	Na(mEq/L)	TSH(mcIU/mL)	Onset of Hyponatremia(day)	HTN	drug	Cell type	Serum osmolarity(mosm/kg)	Urine osmolarity(mosm/kg)	Radom Urine sodium(mmol/L)
**1**	68/F	108	59.29	4	Yes	ARB	papillary	230	252	86
**2**	72/F	114	75.82	3	Yes	ACEi	Papillary	236	90	20
**3**	60/F	115	74.62	2	Yes	HCTZ	Papillary	226	458	129
**4**	64/F	115	72.30	1	No	None	Papillary	237	290	18
**5**	61/F	118	100	1	No	None	papillary	247	228	24
**6**	47/F	119	64.92	1	Yes	ARB	papillary	232	280	18
**7**	62/F	119	100	4	Yes	HCTZ	papillary	N/D	N/D	N/D
**8**	62/F	119	100	3	Yes	HCTZ	follicular	N/D	N/D	N/D
**mean**	**62**	**115.8**	**80.8**	**2.3**						

Abbreviations: TSH, thyroid stimulating hormone; HTN, hypertension; ARB, angiotensin receptor blocker; ACEi angiotensin converting enzyme inhibitor; HCTZ, hydrochlorthiazide; N/D, not done.

### The determinants of hyponatremia following RAI therapy in thyroid cancer patients

We performed univariate and multivariate logistic regression analyses to evaluate the determinants of hyponatremia following RAI therapy in thyroid cancer patients. In univariate analysis, old age, above 60 years, female sex, presence of hypertension, presence of diabetes, thiazide use, use of ARB or ACE inhibitors, lung metastasis, and hyponatremia at the start of RAI were significantly associated with hyponatremia in patients who underwent RAI therapy after total thyroidectomy. Also, lower eGFR, higher calcium, and lower albumin levels in the serum were significantly associated with hyponatremia in thyroid cancer patients following RAI therapy (Table 3). However, in the multivariate analysis, old age, female sex, presence of DM, thiazide use, and hyponatremia at the start of RAI therapy were independently associated with the development of hyponatremia following RAI therapy in thyroid cancer (Table 4).

**Table 3:** Univariate logistic regression analysis showing the association between various parameters and hyponatremia (vs. normonatremia).

	Odds ratio	95% CI	P-value	Overallp-value
Age >60 years (vs. <59 years)				
Moderate to severe	10.063	5.410–18.719	<0.0001	<0.0001
Mild	2.08	1.527–2.833	<0.0001	
Sex (male vs. female)				
Moderate to severe	1.149	0.587–2.248	0.6856	0.0173
Mild	0.615	0.438–0.865	0.0051	
HTN (vs. No HTN)				
Moderate to severe	7.845	4.272–14.407	<0.0001	<0.0001
Mild	1.891	1.375–2.599	<0.0001	
DM (vs. no DM)				
Moderate to severe	2.264	0.874–5.866	0.0925	0.0044
Mild	2.274	1.481–3.492	0.0002	
Thiazide (vs. no use)				
Moderate to severe	9.236	4.476–19.060	<0.0001	<0.0001
Mild	2.780	1.711–4.517	<0.0001	
ARB or ACEi (vs. no use)				
Moderate to severe	3.330	1.614–6.867	0.0011	<0.0001
Mild	2.091	1.442–3.030	<0.0001	
Lung metastasis (vs. no mets)				
Moderate to severe	8.716	2.444–31.082	0.0008	0.0033
Mild	1.842	0.611–5.550	0.2780	
Na level on RAI				
Moderate to severe	0.557	0.486–0.637	<0.0001	<0.0001
Mild	0.74	0.688–0.795	<0.0001	
TSH level on RAI				
Moderate to severe	1.001	0.987–1.016	0.8742	0.3000
Mild	1.005	0.999–1.012	0.1220	
eGFR on RAI				
Moderate to severe	0.979	0.963–0.996	0.0134	0.0186
Mild	0.995	0.989–1.002	0.1424	
eGFR decrease rate				
Moderate to severe	0.996	0.981–1.012	0.6251	0.1621
Mild	1.007	0.999–1.015	0.0740	
Ca on RAI				
Moderate to severe	1.203	0.743–1.946	0.4525	0.0272
Mild	1.323	1.072–1.632	0.0090	
Albumin on RAI				
Moderate to severe	0.273	0.100–0.745	0.0112	0.0034
Mild	0.575	0.364–0.906	0.0172	

Abbreviations: HTN, hypertension; DM, diabetes mellitus; ARB, angiotensin receptor blocker; ACEi angiotensin converting enzyme inhibitor; mets, metastasis.

**Table 4:** Significant predictors ofdeveloping hyponatremia after RAI therapy by multiple logistic regression analysis (vs. normonatremia).

	Odds ratio	95% CI	P-value	Overallp-value
Age >60 years (vs. <59 years)				
Moderate to Severe	8.817	4.296–18.095	<0.0001	<0.0001
Mild	1.875	1.321–2.662	0.0004	
Sex (male vs female)				
Moderate to Severe	0.979	0.456–2.103	0.9575	0.0082
Mild	0.570	0.398–0.814	0.0020	
DM (vs. DM)				
Moderate to Severe	0.732	0.227–2.361	0.6017	0.0653
Mild	1.710	1.048–2.791	0.0318	
Thiazide (vs. no use)				
Moderate to Severe	5.139	1.880–14.045	0.0014	0.0027
Mild	1.900	1.024–3.526	0.0419	
ARB (vs. no use)				
Moderate to Severe	1.597	0.576–4.430	0.3684	0.4277
Mild	0.824	0.500–1.358	0.4476	
Lung metastasis (vs. no mets)				
Moderate to Severe	1.552	0.282–8.548	0.6134	0.8727
Mild	1.052	0.287–3.854	0.9393	
Na on RAI therapy				
Moderate to Severe	0.566	0.488–0.657	<0.0001	<0.0001
Mild	0.726	0.674–0.783	<0.0001	
eGFR on RAI therapy				
Moderate to Severe	0.989	0.974–1.005	0.1715	0.0535
Mild	0.993	0.987–0.999	0.0520	

Abbreviations: DM, diabetes mellitus; ARB, angiotensin receptor blocker; ACEi, angiotensin converting enzyme inhibitor; mets, metastasis; RAI, radioactive iodine; eGFR, estimated glomerular filtration rate.

## Discussion

The primary findings of this study are that old age (especially above 60 years), female sex, thiazide use, and pre-RAI treatment hyponatremia are independent risk factors for developing hyponatremia following RAI therapy in thyroid cancer patients with total thyroidectomy.

These findings were partly consistent with those of previous case reports [8], [9]. In one case report, a 66 year old female patient visited the emergency room and had serum sodium level of 107 mEq/L after low-iodine diet for thyroid ablation, and the author suggested that prolonged low-iodine diet, low salt intake, and use of thiazide diuretics in elderly patients were risk factors for the development of life-threatening hyponatremia [8]. As can be seen in previous case reports [6]–[10], most of the patients were above 60 years of age and all of the patients showed quite severe hyponatremia with serum sodium levels ranging from 107 mEq/L to 121 mEq/L. In contrast to the earlier case reports [6]–[10], in a recent prospective study of 212 patients with DTC [5], 8.5% of the patients had mild hyponatremia (serum sodium level, 130–136 mEq/L) and 1.9% of the patients had moderate to severe hyponatremia (serum sodium level less than 130 mEq/L), and none of the patients had a serum sodium level less than 120 mEq/L. Hence, the authors asserted that clinically-important hyponatremia was uncommon and sodium concentration might not need to be monitored unless the patients had impaired renal function or they were on diuretics in the setting of acute severe hypothyroidism. Our current study also showed that 11.8% of patients had mild hyponatremia (serum sodium level, 131∼137 mEq/L) and only 2.0% of the patients had moderate to severe hyponatremia (serum sodium level less than 130 mEq/L), respectively. These findings support the observation that severe life-threatening hyponatremia did not occur more commonly than expected. However, the finding of 2.0% of patients having moderate to severe hyponatremia should not be considered trivial as the number of thyroid cancer patients are increasing at an alarming rate worldwide and more patients are undergoing RAI treatment. Furthermore, it should not be ignored because all of the 44 patients with moderate to severe hyponatremia in our study showed severe general weakness, nausea or vomiting that necessitated visit to the outpatient clinic or emergency room. Also, in our study, the median age of patients was 47 years, and 76.3% of patients were females. The mean age of the thyroid cancer patients is 46.8 years in Korea [11] and the reported median age at diagnosis of thyroid cancer in the United States of America is 46 years with female predominance [1]. As our data might be representative of the general population of thyroid cancer patients in Korea or United States, our data demonstrated high reliability of the results and solidified risk factors for the development of hyponatremia following RAI therapy in thyroid cancer patients through multivariate analysis in a large number of patients.

In clinical practice, dilutional hyponatremia is a common feature of severe hypothyroidism. The possible mechanisms for the development of hyponatremia in a hypothyroid condition is the inability to excrete a free water load caused by both a decrease in the delivery of water to the distal nephron [12] and excess antidiuretic hormone (ADH) secretion [13]. The glomerular filtration rate is also decreased in hypothyroidism. This can directly diminish free water excretion by diminishing water delivery to the diluting segments [14]. With normal or increased fluid intake, hyponatremia generally results from the inability to decrease urine osmolality below the plasma level failure to suppress maximally the ADH secretion [15]. Due to this mechanism, hypothyroidism may be proposed to be a major risk factor of developing hyponatremia. However, our data showed that hypothyroidism due to thyroid hormone withdrawal was not significantly correlated with post RAI hyponatremia. This finding seems to be consistent with that in another study, which showed that hyponatremia is not necessarily a consequence of hypothyroidism [16]. Taken together, no causal association between hypothyroidism and hyponatremia has been definitively demonstrated. Our current findings suggest there are other risk factors that are associated with development of hyponatremia in post-RAI therapy.

In our study, old age was one of risk factors of hyponatremia following RAI therapy. There is a delay in the ability of the senile kidney to lower sodium excretion in a hyponatremic state [17]. Compared to younger individuals, elderly patients in a hypothyroid state are more susceptible to hyponatremia because of excessive ADH [18], [19]. Therefore, we assumed that elderly patients in a hypothyroid state are at higher risk for hyponatremia compared with younger individuals due to excessive ADH. In addition, use of thiazide diuretics was an independent risk factor for hyponatremia post RAI therapy in our study. The efficacy of ADH depends on the generation of the medullary concentration gradient via NaCl reabsorption in the absence of water, in the thick ascending limb of the loop of Henle. This creates a gradient for water reabsorption via aquaporin-2 insertion in the luminal membranes of the cortical and outer medullary collecting tubules. When hypothyroid patients fail to suppress plasma ADH [13], [20], thiazide acts on the distal tubule, which is not influenced by the medullary concentration gradient, thereby allowing ADH to promote water reabsorption unabated in comparison to loop diuretics [21]–[24]. This may be the reason why thiazide use was associated with hyponatremia following RAI therapy in our study. Also, ARB has a modest natriuretic effect that could induce hyponatremia [25]. In a cross-sectional study of elderly hyponatremic patients presenting to the emergency room, 43.8% of patients were using blockers of the renin-angiotensin system such as ARB or ACEi, some with no associated thiazide diuretic [26]. The risk of hyponatremia should not be underestimated in patients taking ARB. In our study, the use of ARB or ACEi was associated with hyponatremia after RAI therapy on univariate analysis, but when we performed multivariate analysis, the use of ARB or ACE inhibitors lost its statistical validity. Thus, the effect of ARB or ACEi on hyponatremia can be considered insignificant.

Some authorities have indicated that although a low-iodine diet does not mean a low-sodium diet, many patients eat a low-iodine diet with low-sodium diet in spite of the availability of iodine-free salt [6]. Therefore, hyponatremia can be aggravated during a low-iodine diet period. Likewise, in our study, the mean serum sodium level immediately before RAI therapy following prolonged levothyroxine withdrawal was lower than the mean serum sodium level immediately after thyroidectomy (data not shown; 139.7±1.9 mEq/L vs. 140.0±2.1 mEq/L, p = 0.015). Although not stated in the results, severe hyponatremia did not occur immediately before RAI therapy (the lowest serum sodium level at the start of RAI therapy was 126 mEq/L). We observed that the occurrence of severe hyponatremia was less common than we expected before RAI therapy. Thus, this finding suggested that severe hyponatremia occurred more frequently after RAI therapy.

In other case reports [6], [10], because most patients had metastasis to other sites, the authors hypothesized that metastasis might be associated with the development of hyponatremia. Head and neck cancers are responsible for only 1.5% of syndrome of inappropriate secretion of ADH (SIADH) [24] and a rare case report suggested that SIADH can be related to thyroid cancer [27]. In our univariate analysis, we found that lung metastasis had significant association with hyponatremia in moderate to severe group. However, the relationship between lung metastasis and hyponatremia lost its statistical significance on multivariate analysis. Based on our results, lung metastasis is not an independent risk factor for developing hyponatremia following RAI therapy. Therefore, it is difficult to conclude that SIADH due to lung metastasis contributed to the occurrence of hyponatremia in thyroid cancer patients.

Hypothyroidism is also widely accepted as a cause of hypercreatininemia [28]. Baajafer et al. [28] reported that mild elevation in serum creatinine level was common in patients with hypothyroidism. Hammami et al. [5] also showed that hyponatremic patients were more likely to have elevated creatinine concentration. However, our findings suggested that there were no differences on eGFR decrease rate according to the sodium status following RAI therapy. In addition, we did not find a significant association between renal dysfunction at the start of RAI therapy and the occurrence of hyponatremia during RAI therapy on multivariate analysis. These discrepancies cannot be easily explained, but they are attributable to different characteristics and sizes of the study populations.

There are some limitations to our study. First, because the current study was retrospective in nature, we cannot rule out the possibility of residual confounding due to variable selection bias. However, the present study is the largest study to date and it enrolled patients who had similar age or gender characteristics comparable to national registry. Therefore, our data may be representative of general thyroid cancer patients rendering it to compensate for some of the possible bias. Secondly, patients with pseudohyponatremia, dilutional hyponatremia, and polydipsia-induced hyponatremia could not be excluded because serum and urine osmolarity tests were not performed in all of the patients. But, as shown in table 2, in severe hyponatremic patients with available serum and urine osmolarity data, all of the patients showed low serum osmolarity levels. Also, all patients who underwent RAI therapy were clinically stable including well controlled diabetes. Therefore, the possibility of pseudohyponatremia or dilutional hyponatremia could be excluded to some extent. Lastly, as adrenal hormone or volume status in each patient was unavailable, we could not confirm the isolated effect of hypothyroidism in our patients. However, in cases of severe patients admitted to the emergency room, the adrenal hormone values were checked and had none showed severe abnormalities. To the best of our knowledge, this is the first study to systematically quantify the risk factors for the occurrence of hyponatremia following RAI therapy; and thus this study differs from previous case reports [4]–[10] with only limited number of severe hyponatremic cases.

In conclusion, our data suggest that old age, female sex, thiazide medication use, and hyponatremia at the start of RAI are risk factors for developing hyponatremia following RAI therapy after total thyroidectomy. Based on our study results, we recommend that physicians should monitor the occurrence of hyponatremia after RAI therapy in high-risk thyroid carcinoma patients who have undergone total thyroidectomy, and should monitor electrolyte levels closely while the patients should stop taking thiazide diuretics. Excessive hydration can further aggravate hyponatremia in old age patients with electrolyte imbalance. Thus, we recommend the use of isotonic or hypertonic saline instead of sodium-free water drinking or intravenous infusion of hypotonic fluid in order to prevent worsening of hyponatremia. Further, prospective, interventional studies are warranted to evaluate the benefits of discontinuing thiazide prior to RAI therapy in preventing hyponatremia and in improving the clinical outcome, particularly in elderly females.
